# Trends in radiotherapy inpatient admissions in Germany: a population-based study over a 10-year period

**DOI:** 10.1007/s00066-021-01829-7

**Published:** 2021-09-03

**Authors:** Daniel Medenwald, Rainer Fietkau, Gunther Klautke, Susan Langer, Florian Würschmidt, Dirk Vordermark

**Affiliations:** 1 Department of Radiation Oncology, University Hospital Halle (Saale), Ernst-Grube-Str. 40, 06120 Halle (Saale), Germany; 2 Department of Radiation Oncology, Friedrich-Alexander-University Erlangen-Nürnberg, Erlangen, Germany; 3 Department of Radiation Oncology, Chemnitz Hospital, Chemnitz, Germany; 4 Radiological Alliance, Hamburg, Germany

**Keywords:** Inpatient cases, Radiotherapy departments, DRG data, Germany, Non-radiotherapy departments

## Abstract

**Objective:**

With the increasing complexity of oncological therapy, the number of inpatient admissions to radiotherapy and non-radiotherapy departments might have changed. In this study, we aim to quantify the number of inpatient cases and the number of radiotherapy fractions delivered under inpatient conditions in radiotherapy and non-radiotherapy departments.

**Methods:**

The analysis is founded on data of all hospitalized cases in Germany based on Diagnosis-Related Group Statistics (G-DRG Statistics, delivered by the Research Data Centers of the Federal Statistical Office). The dataset includes information on the main diagnosis of cases (rather than patients) and the performed procedures during hospitalization based on claims of reimbursement. We used linear regression models to analyze temporal trends. The considered data encompass the period from 2008 to 2017.

**Results:**

Overall, the number of patients treated with radiotherapy as inpatients remained constant between 2008 (*N* = 90,952) and 2017 (*N* = 88,998). Starting in January 2008, 48.9% of 4000 monthly cases received their treatment solely in a radiation oncology department. This figure decreased to 43.7% of 2971 monthly cases in October 2017. We found a stepwise decrease between December 2011 and January 2012 amounting to 4.3%. Fractions received in radiotherapy departments decreased slightly by 29.3 (95% CI: 14.0–44.5) fractions per month. The number of days hospitalized in radiotherapy departments decreased by 83.4 (95% CI: 59.7, 107.0) days per month, starting from a total of 64,842 days in January 2008 to 41,254 days in 2017. Days per case decreased from 16.2 in January 2008 to 13.9 days in October 2017.

**Conclusion:**

Our data give evidence to the notion that radiotherapy remains a discipline with an important inpatient component. Respecting reimbursement measures and despite older patients with more comorbidities, radiotherapy institutions could sustain a constant number of cases with limited temporal shifts.

**Supplementary Information:**

The online version of this article (10.1007/s00066-021-01829-7) contains supplementary material, which is available to authorized users.

## Introduction

Cancer remains a major health care problem in Europe, with an estimated number of 3.2 million new cases and 1.7 million deaths per year in an ageing population [1, 2].

Radiotherapy is a cornerstone of modern cancer therapy, with half of all cancer patients in Europe receiving radiotherapy at least once [3]. The application of radiotherapy has different objectives, aimed at curative treatment, local control, or palliation [4]. It is one of the most cost-effective cancer treatments and used differently in the different European countries [5]. Germany has the highest number of radiotherapy centers (*n* = 289) in Europe, followed by France (*n* = 177) and Italy (*n* = 172) [3, 6]. Borras et al. calculated that the demand for radiation treatment in Europe will probably increase by about 16% between 2012 and 2025 [7].

In Germany, patients receive inpatient radiotherapy either in radiation oncology departments or in departments with a different specialty where radiotherapy institutions serve as a provider of treatment. This two-arm concept entails important consequences for the field of radiotherapy and health services in oncology.

Various developments in the treatment of cancer patients have taken place in recent years. One trend is the introduction of new treatment options, including new cancer drugs [2].

While most of the treatment in radiotherapy takes place in an outpatient setting, inpatient treatment is a cornerstone when it comes to radiochemotherapy or care of patients in poor health. With more advanced treatments such as immunotherapy that have become essential for the treatment of multiple solid malignancies during recent years, the number of administered cancer drugs has increased and indications have widened [2]. As a result, multimodal concepts have developed [2] and treatment options for older patients have increased considerably, such as in the case of non-small cell lung cancer [7, 8].

A further development is concomitant radiochemotherapy, which, compared to sequential radiochemotherapy, improves overall survival in patients with locally advanced non-small-cell lung cancer (NSCLC) [8] but also in other cancers such as those of the head and neck. Bonner et al. showed that cetuximab in addition to radiotherapy is superior to radiotherapy alone for locally advanced head and neck cancer [9]. Cetuximab was also advantageous when added to concurrent chemotherapy [10], but showed no benefit when it was compared to cisplatin as a single agent [11, 12].

These developments could give rise to a trend where other medical specialties supplement inpatient treatment in radiotherapy departments. Alternatively, an increased demand for inpatient treatment with concomitant radiochemotherapy might lead to increasing case numbers in radiotherapy.

Thus, radiotherapy inpatient departments might lose or gain importance, transforming the field of radiation oncology to a mainly outpatient setting. In this study, we aim to assess and quantify the proportion of inpatients receiving radiotherapy in a genuine radiation oncology department and possible developments since 2008.

## Methods

Our analysis is based on data of all hospitalized cases in Germany as recorded in the Diagnosis-Related Group Statistics (G-DRG Statistics: German version for inpatients) delivered by the Research Data Centers (RDC) of the Federal Statistical Office and the statistical offices of the federal states [13]. Inpatient health care providers use the G‑DRG coding to charge their services and to claim reimbursements. The dataset includes information on the main diagnosis and the procedures performed during the hospitalization. Due to data privacy, the data are based on cases rather than individual patients [4]; that is, the identity of the patient is not coded, only the treated case can be identified. The considered data encompass the period from 2008 (IMRT recorded for the first time) to 2017, and we report them in a monthly pattern (smallest available time unit). We excluded November and December 2017 from the analyses as hospitalizations might well have exceeded the recording period of 2017. As mentioned, the RDC provides the data for public use. Thus, no approval by an ethics committee was required to conduct the analyses (terms outlined by the RDC apply).

Radiotherapeutic procedures were identified by means of the *Operationen- und Prozedurenschlüssel* (OPS). Cases with an OPS code of 8‑522.x (high-voltage radiotherapy, subsequently referred to as “total cases”) were considered for subsequent analyses. Apart from the number of cases, we also report the number of fractions delivered (each coded as a separate procedure in the DRG statistic). The variable “month” as presented here refers to the month of admission. In total, 0.8 million cases entered subsequent analyses.

We defined treatment by means of radiation as encoded by the OPS codes 8‑542 (chemotherapy with minor complexity) and 8‑543 (chemotherapy with medium complexity). In the subsequent text, “radiochemotherapy” refers to radiation and chemotherapy given during one hospital stay.

In a sensitivity analysis, we included all radiotherapy procedures with an OPS code of 8‑520, 8‑521 (orthovolt therapy), 8‑522, 8‑523 (other forms of high-voltage radiotherapy including stereotactic body radiotherapy, total body irradiation), 8‑524 (brachytherapy), and 8‑525 (other forms of brachytherapy including interstitial brachytherapy).

Departments were defined in accordance with the definition of the Federal Statistical Office as structural units within a hospital. Main and sub-departments were regarded as separate entities (e.g., radiotherapy ward/department as part of the oncology department was regarded as separate) [13].

We additionally computed the Charlson Comorbidity Index (CCI) in order to assess the clinical performance status of hospitalized cases.

### Statistics

We used linear regression models to analyze temporal trends, with “month” as the independent variable. In the models reporting hospitalization time, we computed a smoothed trend with respective confidence intervals using the “SSModel” function in R. In the results section we report 95% confidence intervals (CI). All statistical analyses were performed using SAS 9.3 and R (R version 3.6.0, R Core Team (2019). R: A language and environment for statistical computing. R Foundation for Statistical Computing, Vienna, Austria. URL https://www.R-project.org/).

## Results

### Radiotherapy cases

Starting in January 2008, 4000 cases (number per month) received their treatment solely in a radiation oncology department, which decreased to 2971 cases in October 2017. In contrast, there were 4177 cases hospitalized in a non-radiotherapy department in January 2008, decreasing to 3825 at the end of the observational period (annual data in Table 1 and Fig. S7 of the supplement).

**Table 1:** Annual sums (cases and fractions) and the year-specific mean of inpatient days per case

	Cases (*n* per year)				Fractions (*n* per year)				Days/case (mean)	
Year	RT	Both	Non-RT	Sum	RT	Both	Non-RT	Sum	RT	Non-RT
2008	45,144	8872	36,936	90,952	377,963	123,286	267,639	768,888	14.1	19.9
2009	44,669	9020	37,197	90,886	371,493	125,475	264,826	761,794	14.1	20.2
2010	46,494	9117	40,008	95,619	369,585	128,430	286,187	784,202	13.4	19.6
2011	48,545	9247	40,240	98,032	370,287	130,778	291,574	792,639	12.7	19.5
2012	40,406	9503	39,485	89,394	360,399	132,996	282,609	776,004	14.9	19.3
2013	40,144	9532	39,808	89,484	322,768	122,405	256,981	702,154	14.7	19.0
2014	40,795	9918	40,929	91,642	356,164	140,173	292,133	788,470	14.2	18.8
2015	40,949	9257	39,285	89,491	355,807	127,604	279,456	762,867	13.7	18.7
2016	39,216	9626	40,156	88,998	346,900	132,475	288,180	767,555	14.0	18.5

We found a linear decrease in case numbers across the observational period in all cases with radiotherapy considering all departments. This was not true when radiochemotherapy was administered, where we found a trend for an increase in case numbers (0.8 cases per month, 95% CI: 0.1–1.6; Table 2). Consequently, the effect of a decrease was strongest when we considered cases without radiochemotherapy (Fig. S1). The relative decline was most pronounced in the subgroup of cases with radiochemotherapy treated in radiotherapy departments alone (−0.16% per month; 95% CI: −0.2, −0.12).

**Table 2:** Results from linear regression analyses. Values report the absolute change per month or the change in percentage points per year over the observational period

	Radiotherapy units		All units		Proportion (percentage points)
	Absolute	Relative (%)	Absolute	Relative (%)	
*Radiotherapy*					
Hospitalization time	−83.4, (−107, −59.7)	−0.15, (−0.2, −0.11)	−268, (−322.7, −213.3)	−0.22, (−0.26, −0.17)	–
Cases	−6.3, (−7.8, −4.7)	−0.16, (−0.2, −0.12)	−4.4, (−7.1, −1.6)	−0.06, (−0.09, −0.02)	−0.056, (−0.063, −0.048)
Fractions	−29.3, (−44.5, −14)	−0.09, (−0.14, −0.04)	−15.6 (−46.2, 15.1)	−0.02, (−0.07, 0.02)	−0.034, (−0.041, −0.028)
*Radiochemotherapy*					
Hospitalization time	−13.2, (−22.3, −4.1)	−0.08, (−0.13, −0.02)	−41.2, (−59.4, −23.1)	−0.11, (−0.16, −0.06)	–
Cases	0.9, (0.1, 1.6)	0.05, (0.01, 0.09)	1.5, (0.3, 2.8)	0.05, (0.01, 0.09)	0, (−0.008, 0.008)
Fractions	3.4 (−3.4, 10.3)	0.03, (−0.03, 0.08)	10.4, (−2.2, 23)	0.04, (−0.01, 0.1)	−0.009, (−0.017, −0.001)

**Fig. 1:**
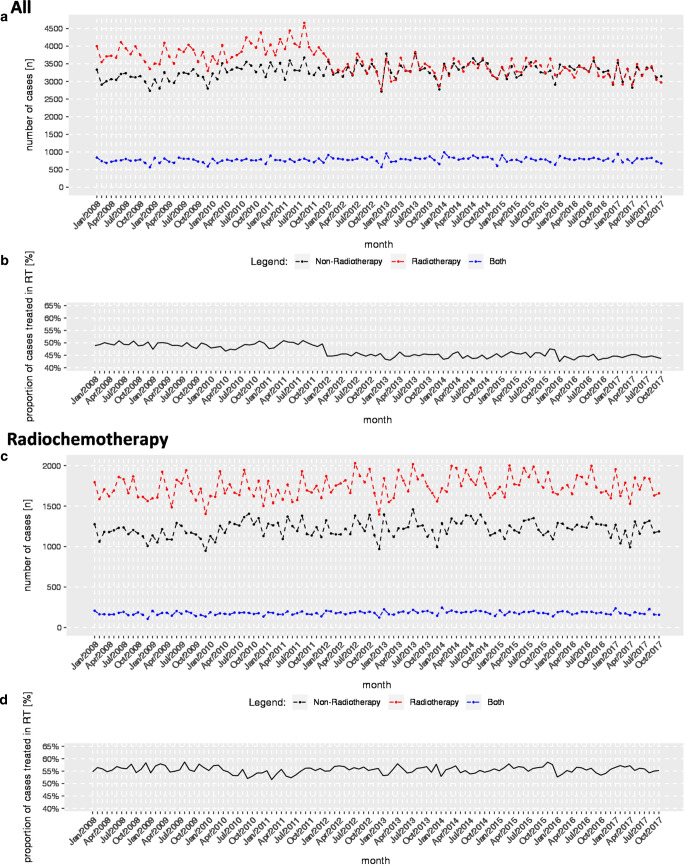
**a** Temporal course of cases with any form of radiotherapy treated in radiotherapy departments (*red*), non-radiotherapy departments (*black*), and other departments (*blue*). **b** Proportion of cases with any form of radiotherapy exclusively treated in radiotherapy departments. **c** Temporal course of cases with radiochemotherapy treated in radiotherapy departments (*red*), non-radiotherapy departments (*black*), and other departments (*blue*). **d** Proportion of cases with radiochemotherapy exclusively treated in radiotherapy departments

Modelling the proportion of cases treated in radiotherapy departments, we found a stepwise decrease between December 2011 and January 2012 amounting to 4.0% (3.3–4.7%; Fig. 1).

### Fractions

**Fig. 2:**
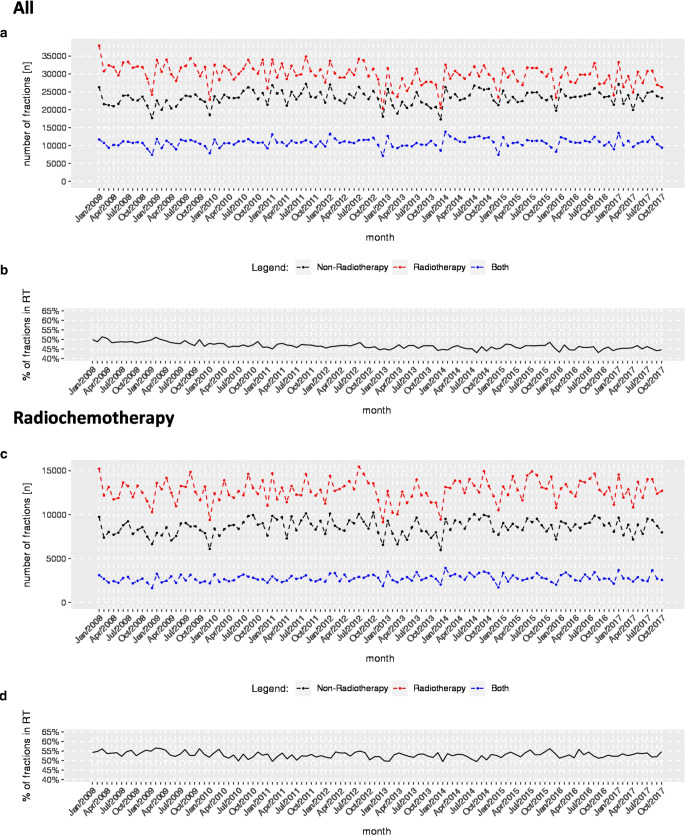
**a** Temporal course of fractions in cases with any form of radiotherapy treated in radiotherapy departments (*red*), non-radiotherapy departments (*black*), and other departments (*blue*). **b** Proportion of cases with any form of radiotherapy exclusively treated in radiotherapy departments. **c** Temporal course of fractions in cases with radiochemotherapy treated in radiotherapy departments (*red*), non-radiotherapy departments (*black*), and other departments (*blue*). **d** Proportion of cases with radiochemotherapy exclusively treated in radiotherapy departments

Similarly, patients hospitalized solely in a radiation oncology department received 37,927 fractions in January 2008, decreasing to 26,310 fractions in October 2017. On average, the linear decrease was 29.3 (95% CI: 14.0–44.5; Fig. 2) fractions per month. Annual summary statistics are shown in Table 1 and in Fig. S7 of the supplement. The only statistically significant decline was found in cases treated in radiotherapy departments considering all radiotherapies (−0.09%; 95% CI −0.14, −0.04; Table 2).

**Fig. 3:**
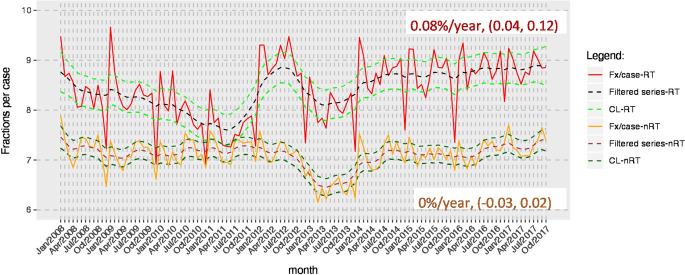
Temporal course of fractions per case differentiating between radiotherapy and non-radiotherapy department, estimates were obtained from linear regression models. *CL* Confidence limits, *n‑RT* non-radiotherapy departments, *RT* radiotherapy departments

In addition, we observed an increase in fractions per case in radiotherapy departments (Fig. 3).

### Days of hospitalization

**Fig. 4:**
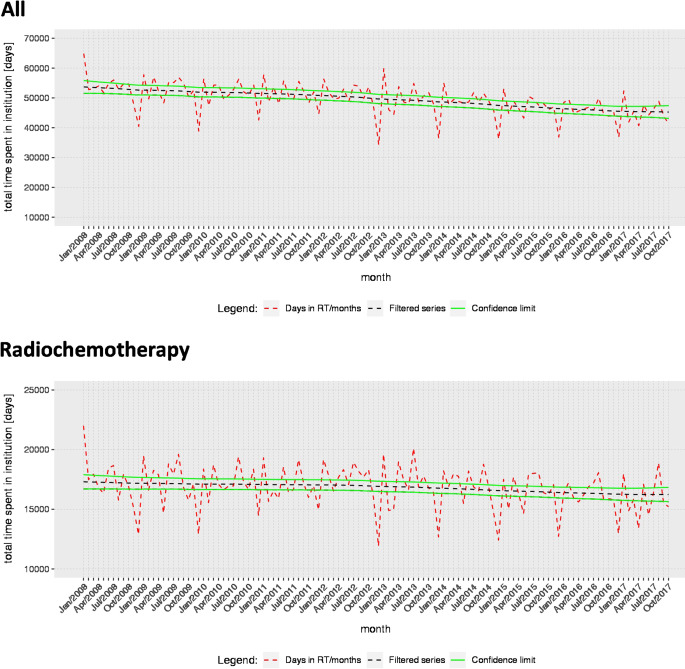
Days of hospitalization in radiotherapy departments differentiating between all radiotherapy and radiotherapy with simultaneous chemotherapy procedures, respectively. Absolute number (*red*), Smoothed temporal course (*black*) with 95% confidence intervals (*green*)

Days hospitalized in radiotherapy departments decreased during the observational period by 83.4 days per month (95% CI: 59.7, 107.0; Fig. 4), starting from a total of 64,842 days in January 2008.

Analyzing the hospitalization time for patients receiving radiochemotherapy in radiotherapy departments, we found a decrease by 13.2 days (95% CI: 4.1, 22.3; Fig. 4) per month during the observational period. In the same line, there was a minor decrease by 41.2 days (95% CI: 23.1, 59.4) when all patients including those treated in a non-radiotherapy department were considered. There was a steep rise in the monthly average of inpatient days per case in a radiotherapy department in January 2012 (Fig. S2).

In relative terms, changes were stronger, with the strongest decrease when all cases were considered, irrespective of the treating department (0.08% per year; Table 2).

### Hospital beds

**Fig. 5:**
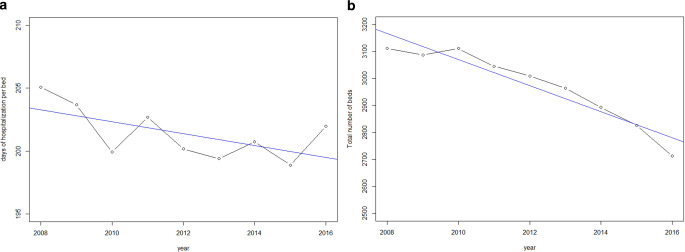
**a** Days hospitalized in a radiotherapy department per bed in Germany, **b** Days hospitalized in a radiotherapy department per bed in Germany (radiochemotherapy only), **c** Sum of beds ascribed to radiotherapy departments in Germany. *Blue line* linear regression line

Hospital beds in radiotherapy departments decreased on average by 51.5% (95% CI: 40.1–63%) per year, starting with 3111 beds in 2008 and falling to 2672 in 2017 (Fig. 5). There was little change in the number of days of hospitalization per bed (0.5 days per bed and year, 95% CI: 0.1–1.0) considering all cases in radiotherapy departments. However, in cases treated with radiochemotherapy, the days spent hospitalized increased by 0.64 days per bed (Fig. 5b).

### Proportion of fractions under radiochemotherapy

**Fig. 6:**
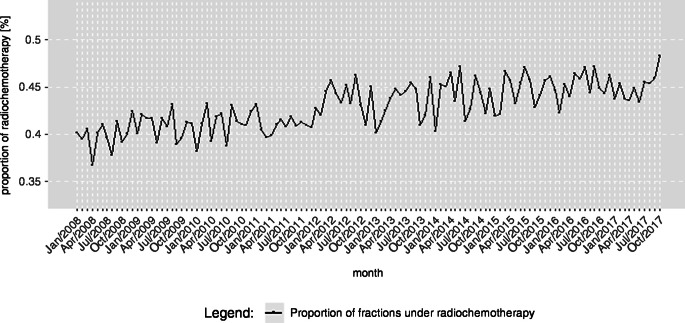
Proportion of cases with chemotherapy in relation to all cases with radiotherapy

When we plotted the proportion of fractions under radio- and chemotherapy during hospitalization, we found a steady increase starting from 40.1% in 2008 and climbing to 48.3% in 2017 (Fig. 6).

### Sensitivity and additional analysis

**Fig. 7:**
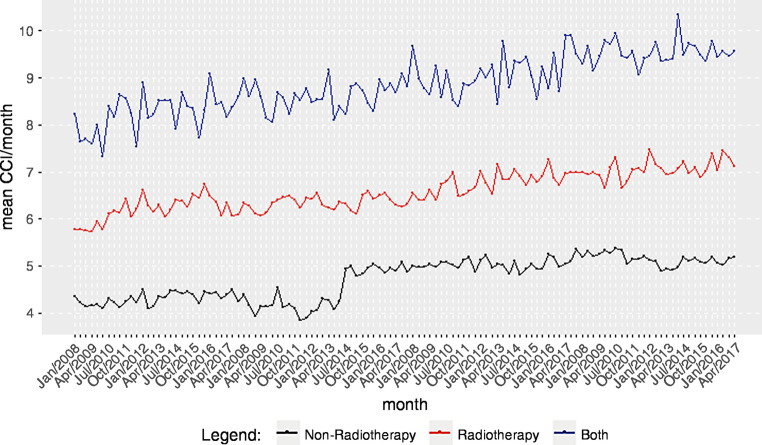
Charlson Comorbidity Index (CCI) in relation to department across the observational period. Radiotherapy departments (*red*), other departments (*black*), and radiotherapy and other departments (*blue*)

The plot of the monthly average of the CCI in relation to department revealed a steady increase in comorbidities of considered cases. Comparing the CCI between departments, we found that the average CCI increased significantly more in radiotherapy than in non-radiotherapy departments (Fig. 7). When we performed the sensitivity analysis of OPS procedures between 8‑521 and 8‑525, we found little change in the computed estimates (Figs. 1, 2 and 3).

## Discussion

Based on German inpatient data, we found that about half of all inpatient cases received their treatment (days of hospitalization and fractions) in genuine radiotherapy departments. In addition, we found a minor decrease in the proportion of cases with treatment in radiotherapy departments from 2008. A decrease in the number of hospital beds accompanied this trend. In addition, patients of all departments had an increasing number of comorbidities. Cases with radiochemotherapy rose only slightly throughout the observational period.

The trend of a slow decrease in total cases of radiotherapy departments, but at least constant numbers in cases receiving radiochemotherapy, might reflect a trend for outpatient treatment. Likewise, the total days of hospitalization decreased constantly throughout the observational period. However, we know little about the development of outpatient treatment in Germany as information are difficult to collect from claims data and are not part of the DRG statistics.

This development is in contrast to a constant number of incident cancer cases since 2008 when all entities are concerned [14]. In the three entities with the highest absolute consumption of radiotherapy according to Wong et al. [15] (breast cancer, prostate cancer, lung cancer), incident cases decreased (prostate, breast) or remained constant (lung in men), but rose in female lung cancer patients. Thus, the observed decreasing trend in case numbers and hospitalization time fails to reflect the overall constant number of newly diagnosed cancer cases in Germany in the observational period.

Our data give evidence to the notion that radiotherapy remains a discipline with an important inpatient component. As treatment becomes more complex and patients become older, radiotherapy clinics could sustained steady case numbers. However, the total amount spent in radiotherapy departments decreased slightly over time, indicating a tendency to shorter hospital stays. These results need to be compared to the small magnitude of the effect. Likewise, as the number of fractions remained constant over time, a possible trend toward hypofractionation and acceleration, as propagated for prostate and breast cancer by recent studies [16, 17], is not present in our data. However, the treatment for both entities will for the most part take place in an outpatient clinic. Considering the inpatient setting, other concepts such as a simultaneous integrated boost instead or accelerated concepts [18], most prominently in the treatment of head and neck cancer [19, 20], might contribute to shorter hospitalizations.

Shorter times of hospitalization went with a decreasing number of hospital beds in radiotherapy departments in Germany. However, hospital beds declined to a similar extent as the total time of hospitalization. Thus, there is no indication of a shortage of treatment capacities in radiotherapy when early years are the reference. This might mitigate the threat of longer waiting times for hospitalization and the initiation of treatment with detrimental effects for the clinical outcome [21]. On the other hand, the number of beds seems to have adapted to a slightly decreasing demand. The fact that case numbers were stable during the entire observational period gives rise to the notion that per admission, cases tend to spend fewer days hospitalized [22]. However, the dip in case numbers between 2011 and 2012 might be due to differences in the DRG coding system when cases with IMRT and conventional external radiotherapy were coded as separate DRGs in 2011, but not in 2012, resulting in fewer recorded admissions in 2011 due to the merger of cases.

Where radiochemotherapy in radiotherapy departments is concerned, we found a minor increase in the number of admitted cases, which was accompanied by a decrease in the number of days spent hospitalized. However, the magnitude of the effect is comparable to the general decline in hospitalization time in all radiotherapy cases, irrespective of the department. Changing treatment patterns avoiding intravenous chemotherapy as in rectal cancer [23] or the establishment of weekly treatment regimens for the treatment of esophageal cancer [24] might contribute. However, such alterations are difficult to estimate as they depend strongly on the entity and particular protocols, as in the case of total neoadjuvant treatment for rectal cancer, where there are protocols with and without additional chemotherapy [25, 26].

Finally, even after the introduction of advanced technologies such as stereotactic radiotherapy, external beam radiotherapy remains the corner stone of inpatient treatment.

Although case numbers decreased more pronouncedly in radiotherapy institutions, the average number of comorbidities increased. Thus, the overall workload of inpatient care might even have increased, with a considerable focus on more complex disorders. Thus, inpatient care in radiotherapy might become more specialized, while treatment of patients in better clinical conditions might move to outpatient care. As cases treated in radiotherapy departments had a higher CCI, which even increased over time, it becomes evident that there is a strong need for inpatient care in radiotherapy. In addition, the treatment landscape might be more selective when in- and outpatient treatment is considered, while a smaller proportion of cases in a worse clinical condition might form the future inpatient cohort. Radiotherapists need to adapt to this change in patient characteristics with, e.g., sufficient quality of multidisciplinary training in the treatment of complex patients.

Until 2011, distinct DRG codes were used for IMRT and conventional 3D radiotherapy in head and neck cancer. The merger of both DRGs in the catalogue might have led to different concepts of inpatient radiotherapy. However, this change in coding failed to affect the number of fractions or hospitalization time.

Related to this fact, the observed steady decrease in the total duration of hospitalization might be due to better ambulatory treatment, especially in the case of palliative care, and less toxic treatments allowing patients to move to an outpatient setting more easily. Outpatient palliative care was introduced in Germany in 2007, which is in line with the start of the observational period in our study.

The development of more specialized treatment facilities might have contributed to fewer cases treated in radiotherapy departments. Therapies such as complex palliative care (*Palliative Komplexbehandlung*), which is reimbursable since 2005 [27], might have shifted cases away from radiotherapy institutions. As a recent survey demonstrated, there is a relevant need for palliative care in radiation oncology [27]. Especially the concept of early palliative care might motivate professionals to favor non-radiotherapy options more frequently [28].

Another contributing factor might be an increase in the number of multidisciplinary oncology departments, making a clear distinction of radiotherapy departments difficult.

In addition, regulatory measures might have an additional effect. However, limits of reimbursement changed sporadically over time [29] and were of small magnitude. Still, the overall effect size of changes in hospital days per case were small in magnitude (within the limits of 1 day per case). Nevertheless, this might not reveal the whole picture of monetary incentives to reduce hospitalization time.

### Limitations

The most important limitation concerns the availability of inpatient data only. Thus, our findings fail to reveal the overall application of radiotherapy in Germany and the interpretation needs to consider this aspect. Thus, we cannot estimate the proportion of patients with treatment in a hospitalized setting. Although we found a decreasing proportion of cases treated in radiotherapy institutions alongside more complex cases, we fail to assess comorbidities and temporal trends of all patients treated by radiotherapy.

Furthermore, the dataset contains cases rather than actual patients. This limitation is especially relevant when re-hospitalization in relation to absolute case numbers is concerned. Here, repetitive admissions of single patients might confound absolute case numbers. Thus, cases of one patient with short intervals between admissions might show up as a single case in the respective claims data. We tried to overcome this limitation by also considering total days of hospitalization, which are not subject to this limitation.

Changes in the DRG recording system might have an additional effect on computed estimates, which might explain the strong decrease in cases between 2011 and 2012.

Cases treated in radiotherapy and non-radiotherapy departments play a minor role in our data, with no apparent change in case numbers. Still, this group might be subject to stronger changes in the near future, when multimodal treatment becomes more established. Furthermore, the data, by showing absolute numbers, report the effect from a provider’s perspective.

In an era of an aging population, the observed trend might even change in the near future, with a higher demand for inpatient radiotherapy [30]. This might lead to an insufficient supply of hospital beds in radiotherapy departments.

## Conclusion

Overall, we observed a decrease in inpatient radiotherapy. Total cases and fractions spent in radiotherapy departments decreased constantly during the observation period. This was also true for total days hospitalized in radiotherapy departments and number of radiotherapy beds.

In conclusion, there is a tendency that inpatient treatment shifts from radiotherapy departments to other disciplines. This has wide-ranging consequences for medical training and education in radiation oncology and the quality of medical care. Even more, the decrease in case numbers might also extend to radiochemotherapy in future years. On the other hand, the increasing severity of cases in terms of comorbidities might reflect a general trend towards more selective and complex inpatient cases. If this is true, we might experience a stronger need for advanced inpatient care focused on patients in poor health along with a wide availability of outpatient institutions to treat patients in a sufficient health condition as appropriate. In either case, the inpatient part of radiotherapy might be subject to important changes to which the field of radiotherapy needs to adapt.

Finally, inpatient cases remain a cornerstone of radiotherapy in Germany. This needs to be added to the general workload for radiation oncologists in Germany [31].

## Supplementary Information

Table S 1: Results from linear regression analyses (OPS 8520–8525). Values report the absolute change per month or the change in percentage points per year over the observational period

Figure S 1: Case numbers of cases with radiotherapy only (without chemotherapy) in terms of department differentiating between all radiotherapy and radiotherapy with simultaneous chemotherapy procedures. A: Temporal course of cases treated in radiotherapy departments (*red*), other departments (*blue*). B: Proportion of cases exclusively treated in radiotherapy departments

Figure S 2: Average inpatient days per case

Figure S 3: Case numbers (OPS: 8520–8525) in terms of department differentiating between all radiotherapy and radiotherapy with simultaneous chemotherapy procedures. A: Temporal course of cases treated in radiotherapy departments (*red*), other departments (*black*), and radiotherapy and other departments (*blue*). B: Proportion of cases exclusively treated in radiotherapy departments

Figure S 4: Numbers of fractions (OPS: 8520–8525) in terms of department differentiating between all radiotherapy and radiotherapy with simultaneous chemotherapy procedures. A: Temporal course of fractions of cases treated in radiotherapy departments (*red*), other departments (*black*), and radiotherapy and other departments (*blue*). B: Proportion of cases exclusively treated in radiotherapy departments

Figure S 5: Days of hospitalization in radiotherapy departments (OPS: 8520–8525) differentiating between all radiotherapy and radiotherapy with simultaneous chemotherapy procedures. Smoothed temporal course (*red*) with 95% confidence intervals (*green*)

Figure S 6: Monthly average Charlson Comorbidity Index (*CCI*) in relation to department

Figure S 7: Numbers of fractions (annual data, OPS: 8520–8525) in terms of department differentiating between all radiotherapy and radiotherapy with simultaneous chemotherapy procedures. A: Temporal course of fractions of cases treated in radiotherapy departments (*red*), other departments (*black*), and radiotherapy and other departments (*blue*). B: Proportion of cases exclusively treated in radiotherapy departments
