# Supplementary material for: Trends in radiotherapy inpatient admissions in Germany: a population-based study over a 10-year period
Source: Strahlenther Onkol. 2021 Sep 3;197(10):865–75. doi: 10.1007/s00066-021-01829-7 (PMC8458212; doi:10.1007/s00066-021-01829-7)
Supplement: Supplementary file 1 — Table S 1: Results from linear regression analyses (OPS 8520–8525). Values report the absolute change per month or the change in percentage points per year over the observational period [file 66_2021_1829_MOESM1_ESM.docx]

| Radiotherapy | Radiotherapy units | All units | Proportion [percentage points] |
| --- | --- | --- | --- |
| Hospitalization time | -82.4, (-107, -57.7) | -316.8, (-378.3, -255.4) |  |
| Cases | -6.8, (-8.5, -5.2) | -6.8, (-9.9, -3.8) | -0.044, (-0.051, -0.036) |
| Fractions | -25.8, (-41.9, -9.6) | -14.7, (-47, 17.5) | -0.028, (-0.034, -0.022) |
| Radiochemotherapy |  |  |  |
| Hospitalization time | -12.5, (-21.6, -3.4) | -57.2, (-77.6, -36.7) |  |
| Cases | 0.9, (0.2, 1.7) | 1.8, (0.5, 3.1) | -0.002, (-0.009, 0.006) |
| Fractions | 3.8, (-3.1, 10.7) | 11.7, (-1, 24.5) | -0.01, (-0.018, -0.001) |
