# Supplementary figures and images for: Trends in radiotherapy inpatient admissions in Germany: a population-based study over a 10-year period
Source: Strahlenther Onkol. 2021 Sep 3;197(10):865–75. doi: 10.1007/s00066-021-01829-7 (PMC8458212; doi:10.1007/s00066-021-01829-7)

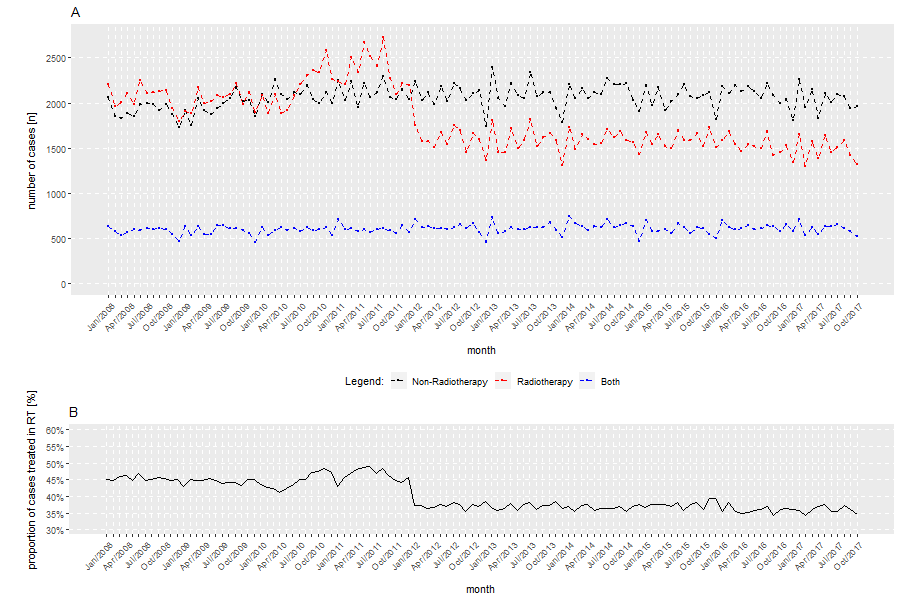

Supplement: Supplementary file 2 — Figure S 1: Case numbers of cases with radiotherapy only (without chemotherapy) in terms of department differentiating between all radiotherapy and radiotherapy with simultaneous chemotherapy procedures. A: Temporal course of cases treated in radiotherapy departments (red), other departments (blue). B: Proportion of cases exclusively treated in radiotherapy departments [file 66_2021_1829_MOESM2_ESM.tiff]

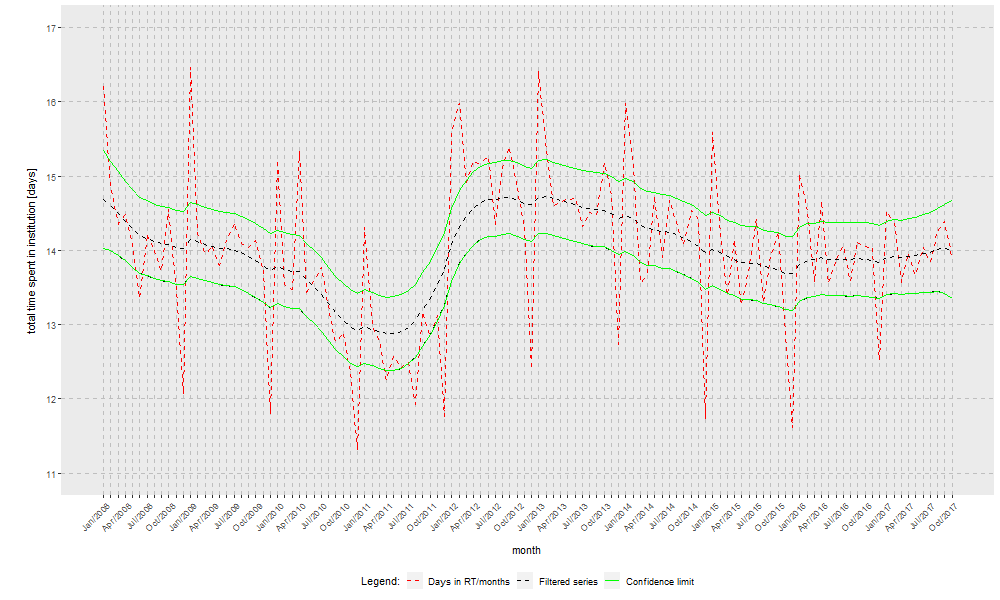

Supplement: Supplementary file 3 — Figure S 2: Average inpatient days per case [file 66_2021_1829_MOESM3_ESM.tiff]

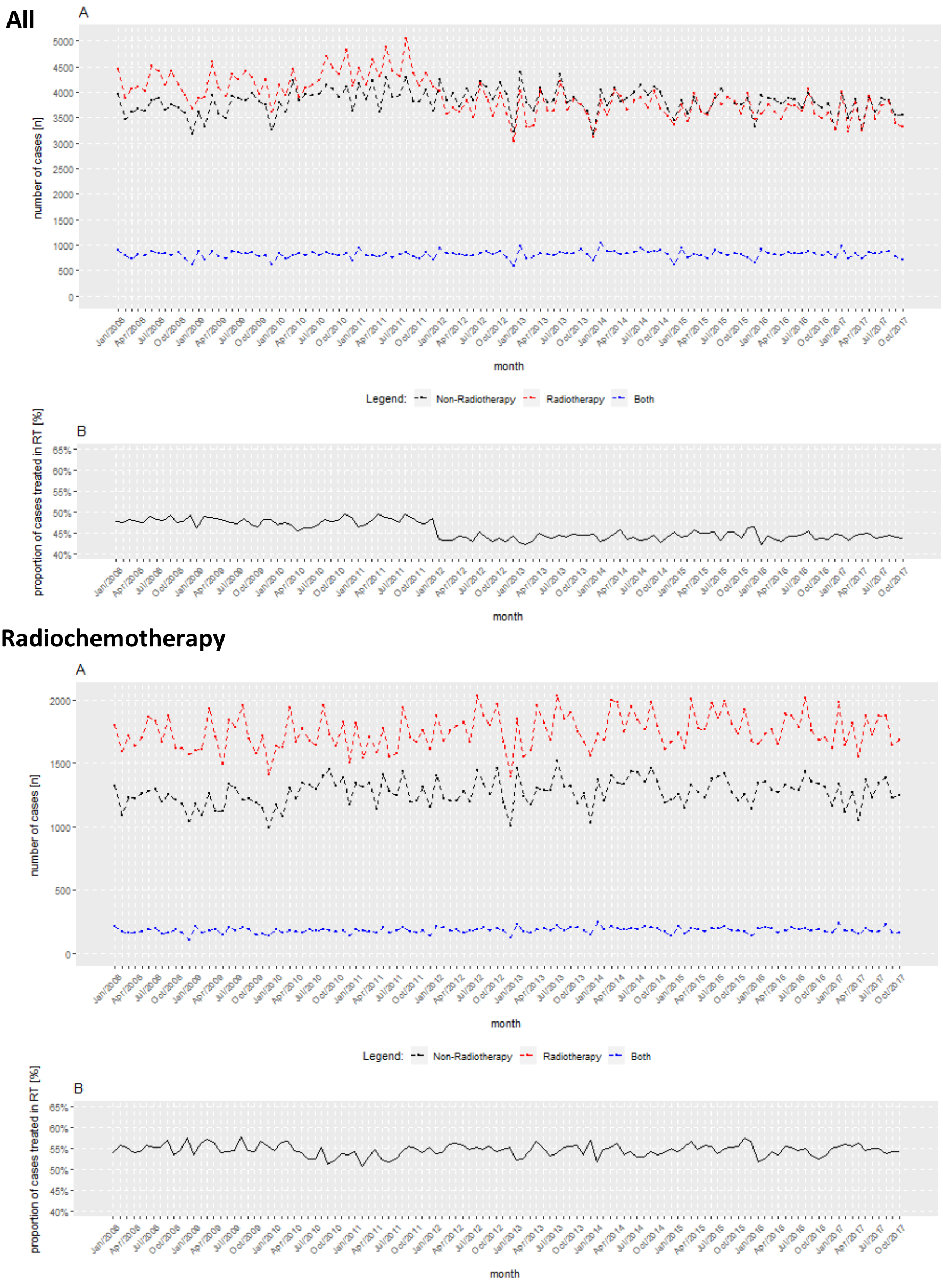

Supplement: Supplementary file 4 — Figure S 3: Case numbers (OPS: 8520–8525) in terms of department differentiating between all radiotherapy and radiotherapy with simultaneous chemotherapy procedures. A: Temporal course of cases treated in radiotherapy departments (red), other departments (black), and radiotherapy and other departments (blue). B: Proportion of cases exclusively treated in radiotherapy departments [file 66_2021_1829_MOESM4_ESM.tif]

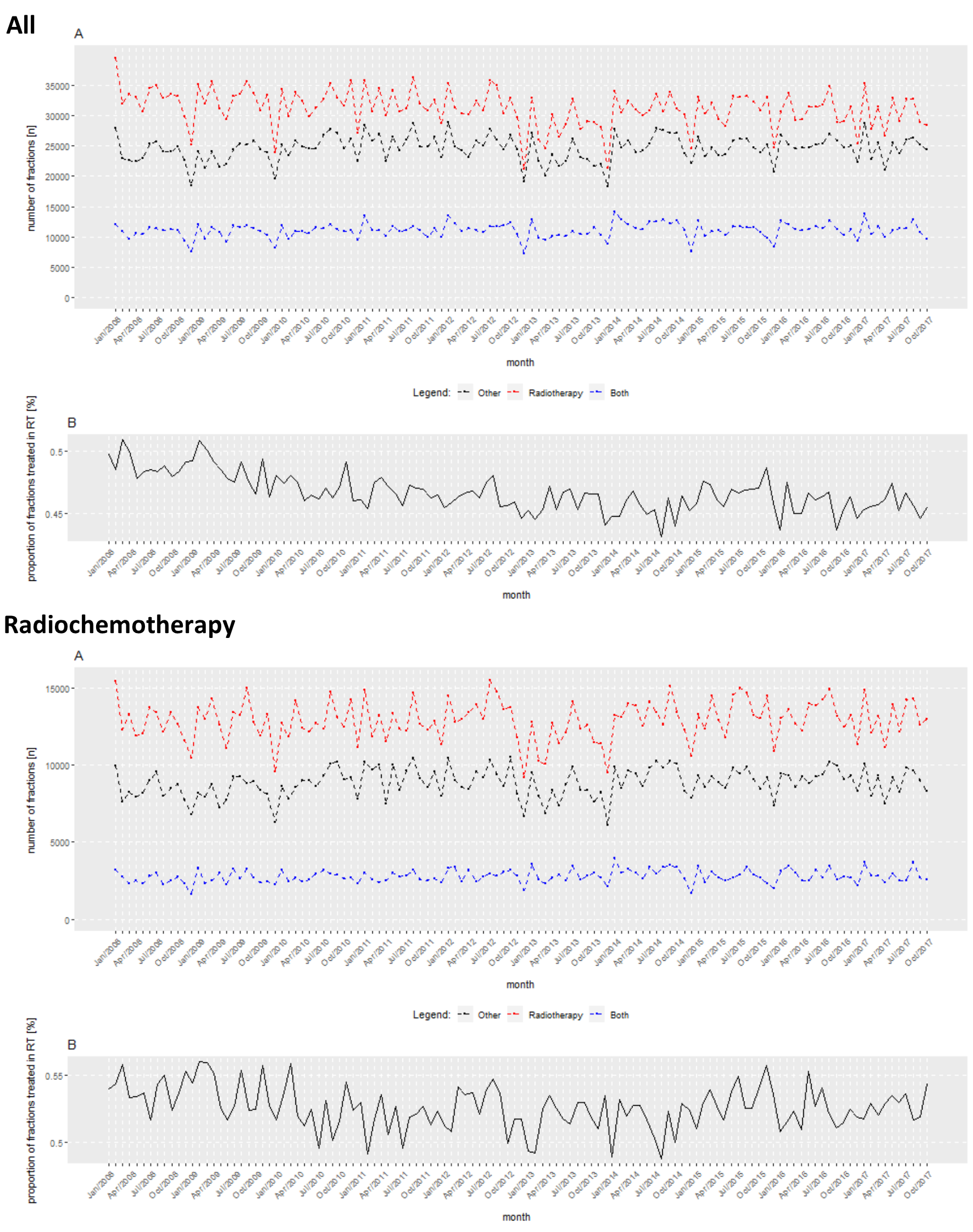

Supplement: Supplementary file 5 — Figure S 4: Numbers of fractions (OPS: 8520–8525) in terms of department differentiating between all radiotherapy and radiotherapy with simultaneous chemotherapy procedures. A: Temporal course of fractions of cases treated in radiotherapy departments (red), other departments (black), and radiotherapy and other departments (blue). B: Proportion of cases exclusively treated in radiotherapy departments [file 66_2021_1829_MOESM5_ESM.tif]

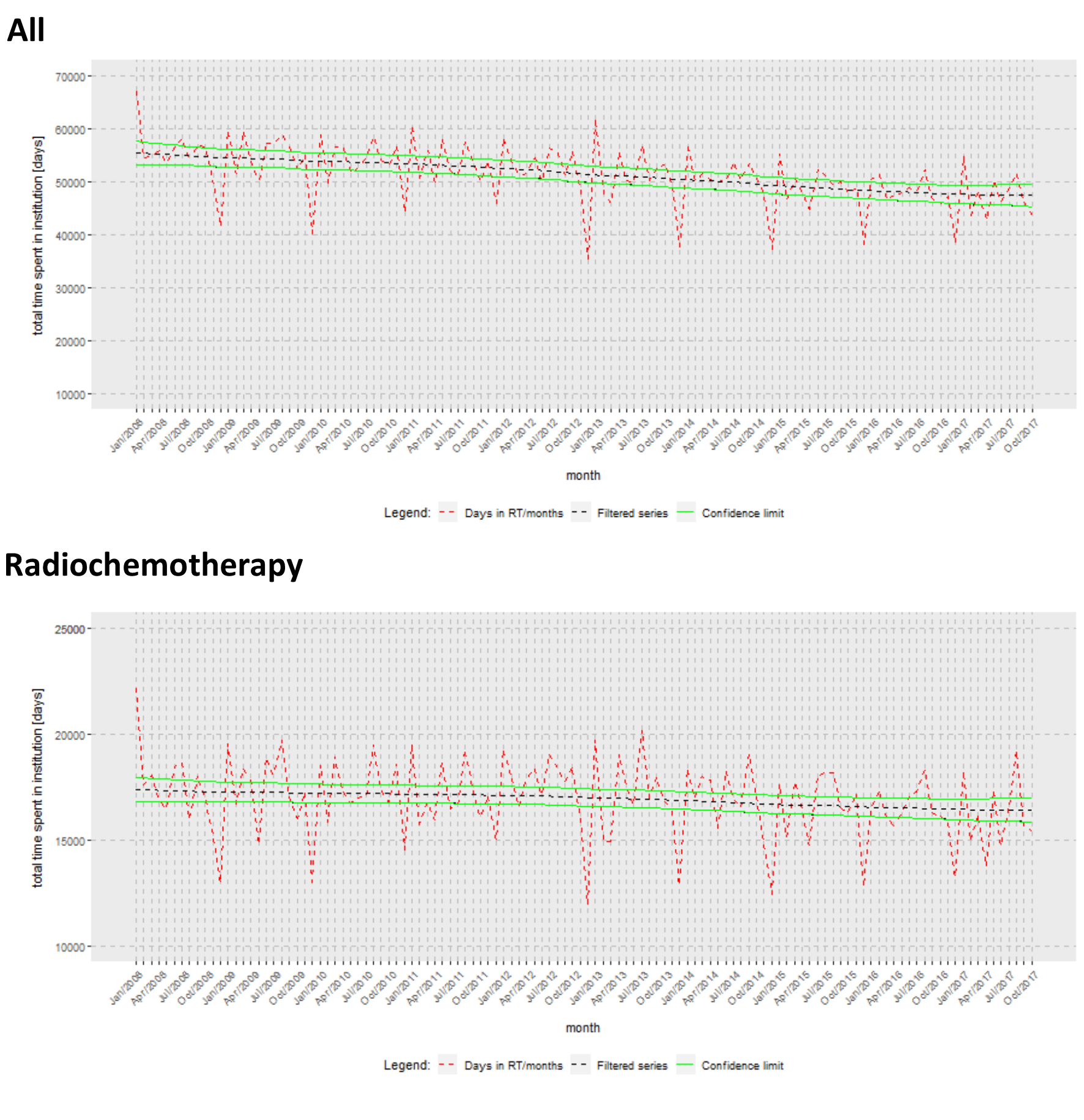

Supplement: Supplementary file 6 — Figure S 5: Days of hospitalization in radiotherapy departments (OPS: 8520–8525) differentiating between all radiotherapy and radiotherapy with simultaneous chemotherapy procedures. Smoothed temporal course (red) with 95% confidence intervals (green) [file 66_2021_1829_MOESM6_ESM.tif]

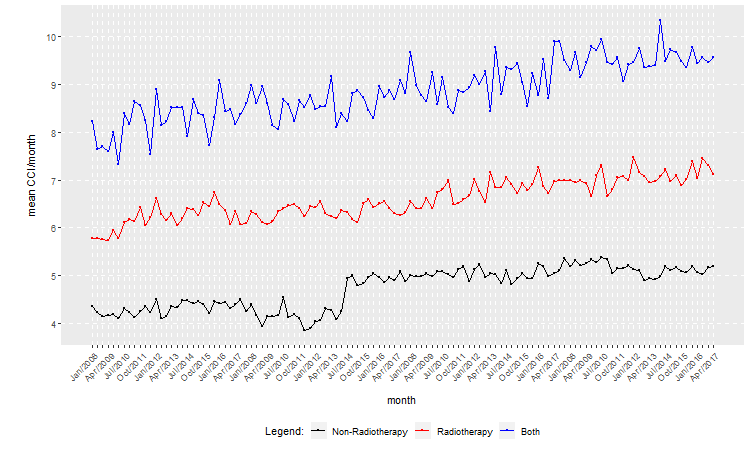

Supplement: Supplementary file 7 — Figure S 6: Monthly average Charlson Comorbidity Index (CCI) in relation to department [file 66_2021_1829_MOESM7_ESM.tiff]

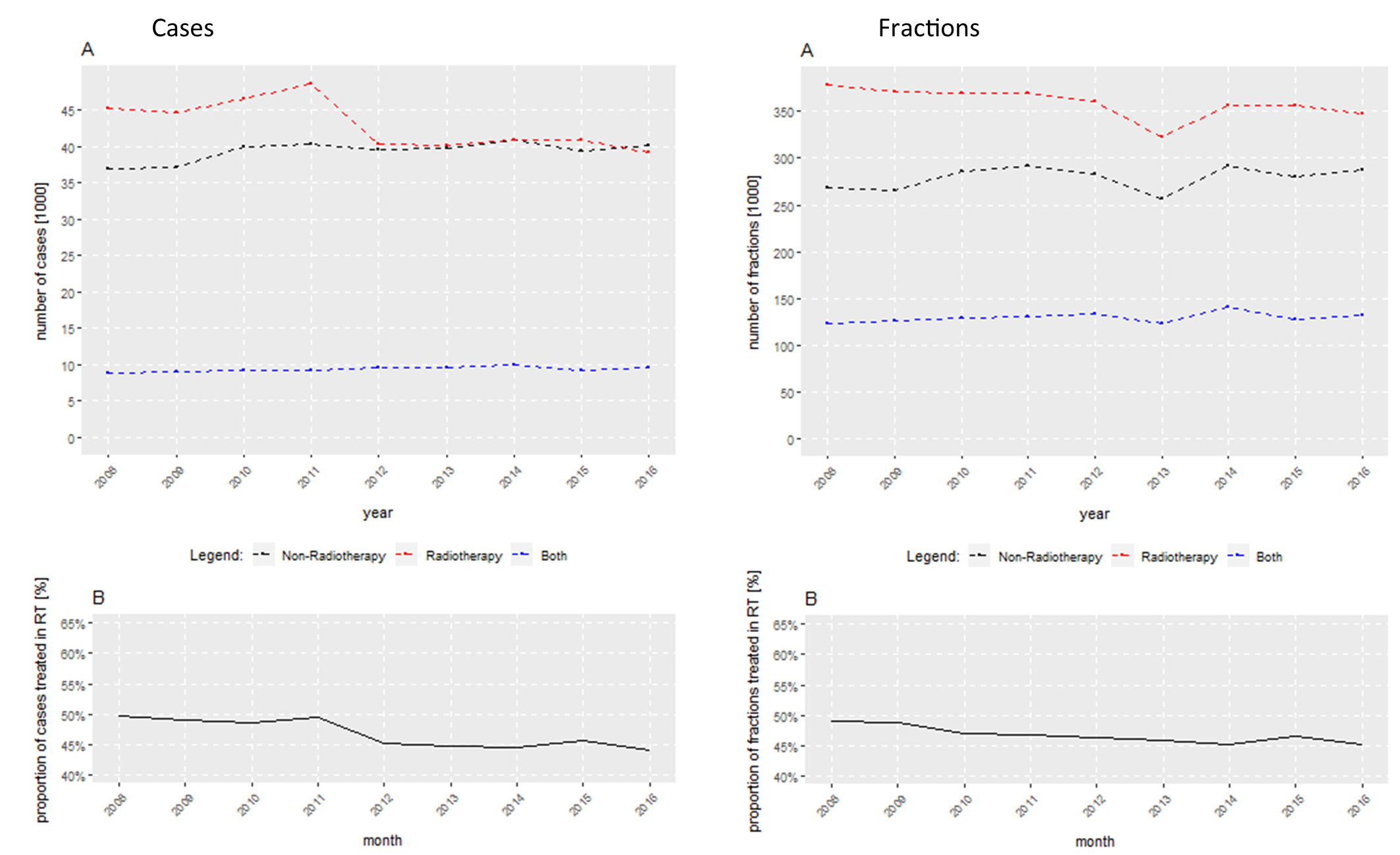

Supplement: Supplementary file 8 — Figure S 7: Numbers of fractions (annual data, OPS: 8520–8525) in terms of department differentiating between all radiotherapy and radiotherapy with simultaneous chemotherapy procedures. A: Temporal course of fractions of cases treated in radiotherapy departments (red), other departments (black), and radiotherapy and other departments (blue). B: Proportion of cases exclusively treated in radiotherapy departments [file 66_2021_1829_MOESM8_ESM.tif]
